# Making brain–machine interfaces robust to future neural variability

**DOI:** 10.1038/ncomms13749

**Published:** 2016-12-13

**Authors:** David Sussillo, Sergey D. Stavisky, Jonathan C. Kao, Stephen I. Ryu, Krishna V. Shenoy

**Affiliations:** 1 Electrical Engineering Department, Stanford University, Stanford, California 94305, USA; 2 Stanford Neurosciences Institute, Stanford, California 94305, USA; 3 Neurosciences Graduate Program, Stanford, California 94305, USA; 4 Palo Alto Medical Foundation, Palo Alto, California 94301, USA; 5 Neurobiology and Bioengineering Departments, Stanford, California 94305, USA; 6 Bio-X Program, Stanford, California 94305, USA; 7 Howard Hughes Medical Institute at Stanford University Stanford University, Stanford, 94305 California, USA

## Abstract

A major hurdle to clinical translation of brain–machine interfaces (BMIs) is that current decoders, which are trained from a small quantity of recent data, become ineffective when neural recording conditions subsequently change. We tested whether a decoder could be made more robust to future neural variability by training it to handle a variety of recording conditions sampled from months of previously collected data as well as synthetic training data perturbations. We developed a new multiplicative recurrent neural network BMI decoder that successfully learned a large variety of neural-to-kinematic mappings and became more robust with larger training data sets. Here we demonstrate that when tested with a non-human primate preclinical BMI model, this decoder is robust under conditions that disabled a state-of-the-art Kalman filter-based decoder. These results validate a new BMI strategy in which accumulated data history are effectively harnessed, and may facilitate reliable BMI use by reducing decoder retraining downtime.

## Abstract (web summary)

Brain-machine interfaces (BMI) depend on algorithms to decode neural signals, but these decoders cope poorly with signal variability. Here, authors report a BMI decoder which circumvents these problems by using a large and perturbed training dataset to improve performance with variable neural signals.

Brain–machine interfaces (BMIs) can restore motor function and communication to people with paralysis^1^,^2^. Progress has been particularly strong towards enabling two-dimensional (2D) computer cursor control, which may allow versatile communications prostheses^3^,^4^,^5^. Cursor-control performance has approached that of the native hand in recent macaque studies^6^,^7^, but this was done under favourable laboratory conditions where neural recordings are often stable both during and across BMI sessions^8^,^9^,^10^,^11^. In contrast to these preclinical studies, one of the major challenges impeding BMI use by human clinical trial participants is the high degree of within- and across-day variability in neural recording conditions (Fig. 1a)^12^,^13^,^14^,^15^,^16^. We use the term ‘recording condition' to broadly encompass the combination of factors that together determine the relationship between observed neural activity and intended kinematics. These factors include the relative position of the electrodes and surrounding neurons (diagrammed in Fig. 1b), variability in sensor properties such as impedance or wiring quality, noise sources and biological factors such as cognitive state or medications. Existing neural decoding algorithms are poorly suited to handle variability in recording condition, resulting in intermittent performance and a need for frequent decoder retraining^4^,^5^,^13^,^17^.

The clinical viability of BMIs would be much improved by making decoders robust to recording condition changes^18^,^19^, and several recent studies have focused on this problem (for example, refs 4, 10, 20, 21, 22, 23, 24, 25, 26, 27, 28, 29). We can broadly divide the conditions that a BMI will encounter into one of two types: (1) conditions that are completely different from what has been previously encountered; and (2) conditions that share some commonality with ones previously encountered. For existing BMI methods, both of these situations necessitate some interruption of function while the decoder is updated to handle the new condition. One strategy for minimizing this interruption is to use adaptive decoders, which update their parameters based on new data collected during the BMI's use (rather than collecting new training data for a *de novo* decoder) to try to better match the new recording condition^4^,^10^,^20^,^21^,^22^,^23^,^24^,^25^,^26^,^27^,^28^,^29^. In the first case, this is likely the best that can be done. But in the second case, BMI interruption could in principle be avoided altogether by a decoder capable of exploiting the similarities between the current and previously encountered conditions (Fig. 1c).

We were motivated to try this complimentary strategy because chronic BMI systems do typically encounter recording conditions in which there is some commonality with past recording conditions^8^,^10^,^13^,^14^,^27^,^28^,^30^,^31^,^32^. Furthermore, these systems generate and store months, or even years, of neural and kinematic data as part of their routine use. Almost all of these past data are left unused in existing BMI systems: decoders are trained using the most recently available data, typically from a block of calibration trials at the start of that day's experiment, or from a recent previous experiment^33^. Using this historical data would be difficult for most BMI decoders, as they are linear (for example, refs 2, 6). Linear decoders are prone to underfitting heterogeneous training sets, such as those that might be sampled from months of data. To overcome this limitation, an essential aspect of our approach is to use a nonlinear and computationally ‘powerful' decoder (that is, one capable of approximating any complex, nonlinear dynamical system), which should be capable of learning a diverse set of neural-to-kinematic mappings.

Specifically, we tested whether one could gain traction on the decoder robustness problem by exploiting this idle wealth of stored data using an artificial recurrent neural network (RNN). We did this with a three-pronged approach. The first was the use of the nonlinear RNN. The second was to train the decoder from many months of previously recorded data. Third, to ‘harden' the decoder against being too reliant on any given pattern of inputs, we artificially injected additional variability into the data during decoder training.

The fact that conventional state-of-the-art decoding methods, which tend to be linear or at least of limited computational complexity^34^, work well for closed-loop BMI control of 2D cursors demonstrates that the model mismatch of assuming linear neural-to-kinematic mappings is well tolerated for a given recording condition. Nevertheless, when neural-to-kinematic mappings change over time, a conventional decoder trained on many days' data is almost certainly not going to fully benefit from this abundance of the data. This is because it requires a nonlinear algorithm to learn a set of different context-dependent mappings, even if these individual mappings from neural firing rates to kinematics were entirely linear (which they are not). Methods such as linear Kalman filters can at best only learn an average mapping, ‘splitting the difference' to reduce error across days in the training set. This approach is not well-suited for most of the recording conditions. We therefore developed a new BMI decoder using a nonlinear RNN variant called the multiplicative recurrent neural network (MRNN) developed by Sutskever and colleagues^35^ using their Hessian-free technique for training RNNs^36^. Several properties of the MRNN architecture, which was originally used for character-level language modelling, make it attractive for this neural prosthetic application. First, it is recurrent, and can therefore ‘remember' state across time (for example, during the course of a movement), potentially better matching the time-varying, complex relationships between neural firing rates and kinematics^37^,^38^. Second, its ‘multiplicative' architecture increases computational power by allowing the neural inputs to influence the internal dynamics of the RNN by changing the recurrent weights (Fig. 2a). Loosely speaking, this allows the MRNN to learn a ‘library' of different neural-to-kinematic mappings that are appropriate to different recording conditions. The MRNN was our specific choice of nonlinear method for learning a variety of neural-to-kinematic mappings, but this general approach is likely to work well with many out-of-the-box RNN variants, such as a standard RNN (for example, ref. 38) or LSTM^39^. Our approach is also completely complementary to adaptive decoding.

We evaluated decoders using two non-human primates implanted with chronic multielectrode arrays similar to those used in ongoing clinical trials. We first show that training the MRNN with more data from previous recording sessions improves accuracy when decoding new neural data, and that a single MRNN can be trained to accurately decode hand reach velocities across hundreds of days. We next present closed-loop results showing that an MRNN trained with many days' worth of data is much more robust than a state-of-the-art Kalman filter-based decoder (the Feedback Intention Trained Kalman filter, or FIT-KF^40^) to two types of recording condition changes likely to be encountered in clinical BMI use: the unexpected loss of signals from highly-informative electrodes, and day-to-day changes. Finally, we show that this robustness does not come at the cost of reduced performance under more ideal (unperturbed) conditions: in the absence of artificial challenges, the MRNN provides excellent closed-loop BMI performance and slightly outperforms the FIT-KF. To our knowledge, this is the first attempt to improve robustness by using a large and heterogeneous training dataset: we used roughly two orders of magnitude more data than in previous closed-loop studies.

## Results

### MRNN performance improves with more data

**Figure 2:**
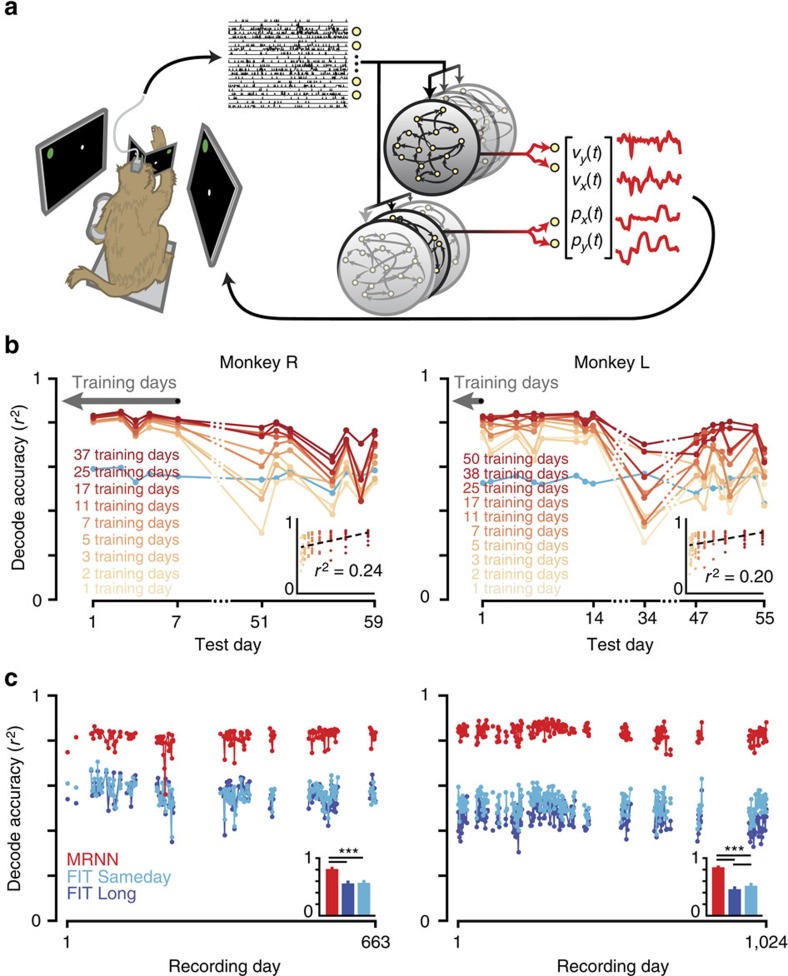
An MRNN decoder can harness large training data sets. (**a**) A monkey performed a target acquisition task using his hand while multiunit spikes were recorded from multielectrode arrays in motor cortex. Data from many days were used to train two MRNNs such that velocity and position were read out from the state of their respective internal dynamics. These MRNN internal dynamics are a function of the binned neural spike counts; thus, the MRNN can conceptually be thought of as selecting an appropriate decoder at any given time based on the neural activity. (**b**) We evaluated each MRNN's ability to reconstruct offline hand velocity on 12 (16) monkey R (L) test days after training with increasing numbers of previous days' data sets. Training data were added by looking further back in time so as to not conflate training data recency with data corpus size. In monkey R, early test days also contributed training data (with test trials held out). In monkey L, from whom more suitable data was available, the training data sets started with the day prior to the first test day. More training data (darker coloured traces) improved decode accuracy, especially when decoding more chronologically distant recording conditions. We also plotted performance of a FIT Kalman filter trained from each individual day's training data (‘FIT Sameday', light blue). (Insets) show the same MRNN data in a scatter plot of decode accuracy versus number of training days (99 data points for monkey R, 160 for L). Linear fit trend lines reveal a significant positive correlation. (**c**) An MRNN (red trace) was trained with data from 154 (250) monkey R (L) recording days spanning many months. Its offline decoding accuracy on held-out trials from each of these same days was compared with that of the FIT Sameday (light blue). We also tested a single FIT-KF trained using the same large dataset as the MRNN (‘FIT Long', dark blue). Gaps in the connecting lines denote recording gaps of more than ten days. (Insets) mean±s.d. decode accuracy across all recording days. Stars denote *P<*0.001 differences (signed-rank test). The MRNN outperformed both types of FIT-KF decoders on every day's dataset.

We first tested whether training the MRNN with many days' worth of data can improve offline decoder performance across a range of recording conditions. This strategy was motivated by our observation that the neural correlates of reaching—as recorded with chronic arrays—showed day-to-day similarities (Supplementary Fig. 1). For a typical recording session, the most similar recording came from a chronologically close day, but occasionally the most similar recording condition was found in chronologically distant data. MRNN decoders were able to exploit these similarities: Figure 2b shows that as more days' data (each consisting of ∼500 point to point reaches) were used to train the decoder, the accuracy of reconstructing reach velocities, measured as the square of the Pearson's correlation coefficient between true and decoded test data set velocity, increased (positive correlation between number of training days and decoded velocity accuracy, *r*^2^=0.24, *P*=2.3e−7 for monkey R (*n*=99), *r*^2^=0.20, *P*=3.2e−9 for monkey L (*n*=160), linear regression). In particular, these results show that using more training data substantially increased the decode accuracy for the ‘hard' days that challenged decoders trained with only a few days' data (for example, test day 51 for monkey R). Further, this improvement did not come at the cost of worse performance on the initially ‘easy' test days. These results demonstrate that larger training data sets better prepare the MRNN for a variety of recording conditions, and that learning to decode additional recording conditions did not diminish the MRNN's capability to reconstruct kinematics under recording conditions that it had already ‘mastered'. There was not a performance versus robustness trade-off.

We then tested whether the MRNN's computational capacity could be pushed even further by training it using the data from 154 (250) different days' recording sessions from monkey R (L), which spanned 22 (34) months (Fig. 2c). The MRNN's offline decode accuracy was *r*^2^=0.81±0.04 (mean±s.d., monkey R) and *r*^2^=0.84±0.03 (monkey L) across all these recording sessions' held-out test trials. For comparison, we tested the decode accuracy of the FIT-KF trained in two ways: either specifically using reaching data from that particular day (‘FIT Sameday'), or trained on the same large multiday training data set (‘FIT Long'). Despite the multitude of recording conditions that the MRNN had to learn, on every test day each monkey's single MRNN outperformed that day's FIT Sameday filter (monkey R (*n*=154 samples): FIT Sameday *r*^2^=0.57±0.05, *P*=1.2e−153 signed-rank test comparing all days' FIT Sameday and MRNN *r*^2^; monkey L (*n*=250 samples): *r*^2^=0.52±0.05, *P*=2.1e−319). Unsurprisingly, a linear FIT-KF did not benefit from being trained with the same large multiday training set and also performed worse than the MRNN (monkey R: FIT Long *r*^2^=0.56, *P*=5.1e−27 comparing all days' FIT Long to MRNN r^2^; monkey L: *r*^2^=0.46±0.05, *P*=9.3e−43).

While these offline results demonstrate that the MRNN can learn a variety of recording conditions, experiments are required to evaluate whether this type of training leads to increased decoder robustness under closed-loop BMI cursor control. In closed-loop use, the BMI user updates his or her motor commands as a result of visual feedback, resulting in distributions of neural activity that are different than that of the training set. Thus, results from offline simulation and closed-loop BMI control may differ^32^,^41^,^42^,^43^. To this end, we next report closed-loop experiments that demonstrate the benefit of this training approach.

### Robustness to unexpected loss of informative electrodes

We next performed closed-loop BMI cursor-control experiments to test the MRNN's robustness to recording condition changes. The first set of experiments challenged the decoder with an unexpected loss of inputs from multiple electrodes. The MRNN was trained with a large corpus of hand-reaching training data up through the previous day's session (119–129 training days for monkey R, 212–230 days for monkey L). Then, its closed-loop performance was evaluated on a Radial 8 Task, while the selected electrodes' input firing rates were artificially set to zero. By changing how many of the most informative electrodes were dropped (‘informative' as determined by their mutual information with reach direction; see Methods), we could systematically vary the severity of the challenge. Since this experiment was meant to simulate sudden failure of electrodes during BMI use (after the decoder had already been trained), we did not retrain or otherwise modify the decoder based on knowledge of which electrodes were dropped. There were no prior instances of these dropped electrode sets having zero firing rates in the repository of previously collected training data (Supplementary Fig. 2). Thus, this scenario is an example of an unfamiliar recording condition (zero firing rates on the dropped electrodes) having commonality with a previously encountered condition (the patterns of activity on the remaining electrodes).

**Figure 3:**
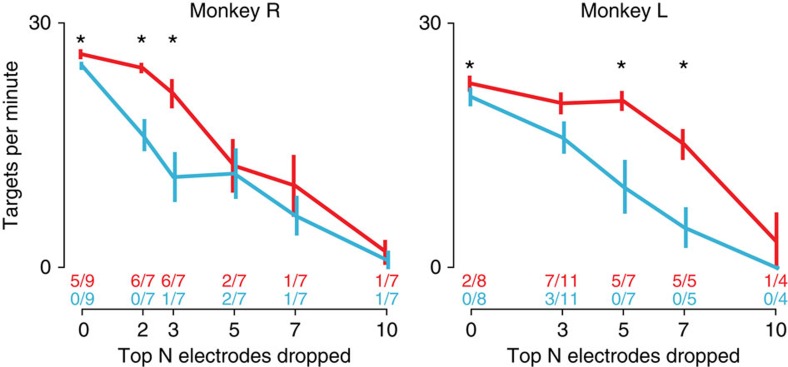
Robustness to unexpected loss of the most important electrodes. Closed-loop BMI performance using the MRNN (red) and FIT Sameday (blue) decoders while simulating an unexpected loss of up to 10 electrodes by setting the firing rates of these electrodes to zero. The mean and s.e.m. across experimental sessions' targets per minute performance is shown for each decoder as a function of how many electrodes were removed. Stars denote conditions for which the MRNN significantly outperformed FIT Sameday across sessions (*P*<0.05, paired *t*-test). The text above each condition's horizontal axis tick specifies for how many of the individual evaluation days MRNN (red fraction) or FIT Sameday (blue fraction) performed significantly better than the other decoder according to single-session metrics of success rate and time to target. Electrode-dropping order was determined by the mutual information between that electrode's spike count and target direction during arm-controlled reaches.

We found that the MRNN was robust to severe electrode-dropping challenges. It suffered only a modest loss of performance after losing up to the best 3 (monkey R) or 5 (monkey L) electrodes (Fig. 3). We compared this with the electrode-dropped performance of a FIT-KF decoder trained with hand-reaching calibration data from the beginning of that day's experiment^6^,^40^ (‘FIT Sameday') by alternating blocks of MRNN and FIT Sameday control in an ‘AB AB' interleaved experiment design. FIT Sameday decoder's performance worsened markedly when faced with this challenge. Across all electrode-dropped conditions, Monkey R acquired 52% more targets per minute using the MRNN, while Monkey L acquired 92% more targets. Supplementary Movie 2 shows a side-by-side comparison of the MRNN and FIT Sameday decoders with the three most informative electrodes dropped.

Although the past data sets used to train the MRNN never had these specific sets of highly important electrodes disabled, our technique of artificially perturbing the true neural activity during MRNN training did generate training examples with reduced firing rates on various electrodes (as well as examples with increased firing rates). The MRNN had therefore been broadly trained to be robust to firing rate reduction on subsets of its inputs. Subsequent closed-loop comparisons of MRNN electrode-dropping performance with and without this training data augmentation confirmed its importance (Supplementary Fig. 3a). An additional offline decoding simulation, in which MRNN decoders were trained with varying data set sizes with and without training data augmentation, further shows that both the MRNN architecture and its training data augmentation are important for robustness to electrode dropping (Supplementary Fig. 4). These analyses also suggest that when data augmentation is used, large training data set size does not impart additional robustness to these particular recording condition changes. This is not surprising given that the previous data sets did not include examples of these electrodes being dropped.

### Robustness to naturally sampled recording condition changes

The second set of closed-loop robustness experiments challenged the MRNN with naturally occurring day-to-day recording condition changes. In contrast to the highly variable recording conditions encountered in human BMI clinical trials, neural recordings in our laboratory set-up are stable within a day and typically quite stable on the time scale of days (Supplementary Fig. 2; ref. 10). Therefore, to challenge the MRNN and FIT-KF decoders with greater recording condition variability, we evaluated them after withholding the most recent several months of recordings from the training data. We refer to this many-month interval between the most recent training data day and the first test day as the training data ‘gap' in these ‘stale training data' experiments. The gaps were chosen arbitrarily within the available data, but to reduce the chance of outlier results, we repeated the experiment with two different gaps for each monkey.

For each gap, we trained the MRNN with a large data set consisting of many months of recordings preceding the gap and compared it with two different types of FIT-KF decoders. The ‘FIT Old' decoder was trained from the most recent available training day (that is, the day immediately preceding the gap); this approach was motivated under the assumption that the most recent data were most likely to be similar to the current day's recording condition. The ‘FIT Long' decoder was trained from the same multiday data set used to train the MRNN and served as a comparison in which a conventional decoder is provided with the same quantity of data as the MRNN. The logic underlying this FIT Long approach is that despite the Kalman filter being ill-suited for fitting multiple heterogeneous data sets, this ‘averaged' decoder might still perform better than the FIT Old trained using a single distant day.

**Figure 4:**
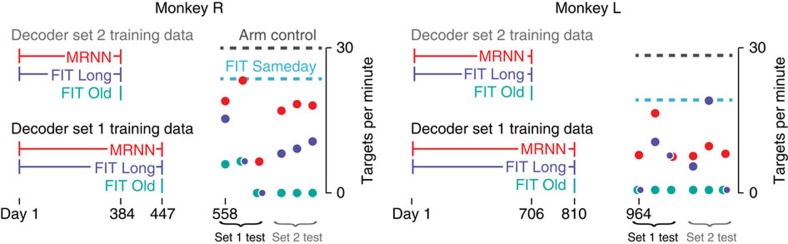
Robustness to naturally occurring recording condition changes. We created decoder evaluation conditions in which the neural inputs were likely to be different from much of the training data by withholding access to the most recent several months of data. Each circle corresponds to the mean closed-loop BMI performance using these ‘stale' MRNN (red), FIT Long (dark blue) and FIT Old (teal) decoders when evaluated on six different experiment days spanning 7 (13) days in monkey R (L). Each test day, these three decoders, as well as a FIT Sameday decoder trained from that day's arm reaches, were evaluated in an interleaved block design. The legend bars also denote the time periods from which training data for each stale decoder came from. We repeated the experiments for a second set of decoders to reduce the chance that the results were particular to the specific training data gap chosen. The training data periods contained 82 and 92 data sets (monkey R) and 189 and 200 training data sets (monkey L). The only decoder that was consistently usable, that is, did not fail on any test days, was the MRNN. To aid the interpretation of these stale decoder performances, we show the average performance across the six experiment days using arm control (grey dashed line) or a FIT Sameday decoder (blue dashed line).

We found that the MRNN was the only decoder that was reliably usable when trained with stale data (Fig. 4). FIT Old performed very poorly in both monkeys, failing completely (defined as the monkey being unable to complete a block using the decoder, see Methods) in 4/6 monkey R experimental sessions and 6/6 monkey L sessions. FIT Long performed better than FIT Old, but its performance was highly variable—it was usable on some test days but failed on others. In Monkey R, the across-days average acquisition rate was 105% higher for the MRNN than FIT Long (*P*=4.9e−4, paired *t*-test). Monkey L's MRNN did not perform as consistently well as Monkey R's, but nevertheless demonstrated a trend of outperforming FIT Long (32% improvement, *P*=0.45), in addition to decidedly outperforming FIT Old, which failed every session. Although monkey L's FIT Long outperformed the MRNN on one test day, on all other test days FIT Long was either similar to, or substantially worse than, MRNN. Moreover, whereas the MRNN could be used to control the cursor every day, FIT Long was not even capable of acquiring targets on some days. Further tests of additional FIT Old decoders confirmed that they generally perform poorly (Supplementary Fig. 5). The lack of consistent usability by any of the FIT-KF decoders (Old or Long) demonstrates that having access to a large repository of stale training data does not enable training a single Kalman filter that is robust to day-to-day variability in recording conditions. In contrast, an MRNN trained with this large data set was consistently usable.

To further demonstrate the consistency of these results, we performed offline simulations in which we tested MRNN decoders on additional sets of training and test data sets separated by a gap. Each set was non-overlapping with the others, and together they spanned a wide range of each animal's research career. We observed the same trends in these offline simulations: MRNNs trained with many previous days of training data outperformed FIT Old and FIT Long decoders (Supplementary Fig. 6). In these analyses, we also dissected which components of our decoding strategy contributed to the MRNN's robustness. We did this by comparing MRNNs trained with varying numbers of days preceding the gap, with or without training data spike rate perturbations. The results show that training using more data, and to a lesser extent incorporating data augmentation (see also closed-loop comparisons in Supplementary Fig. 3b), contributed to the MRNN's robustness to naturally occurring recording condition changes.

### High-performance BMI using the MRNN decoder

**Figure 5:**
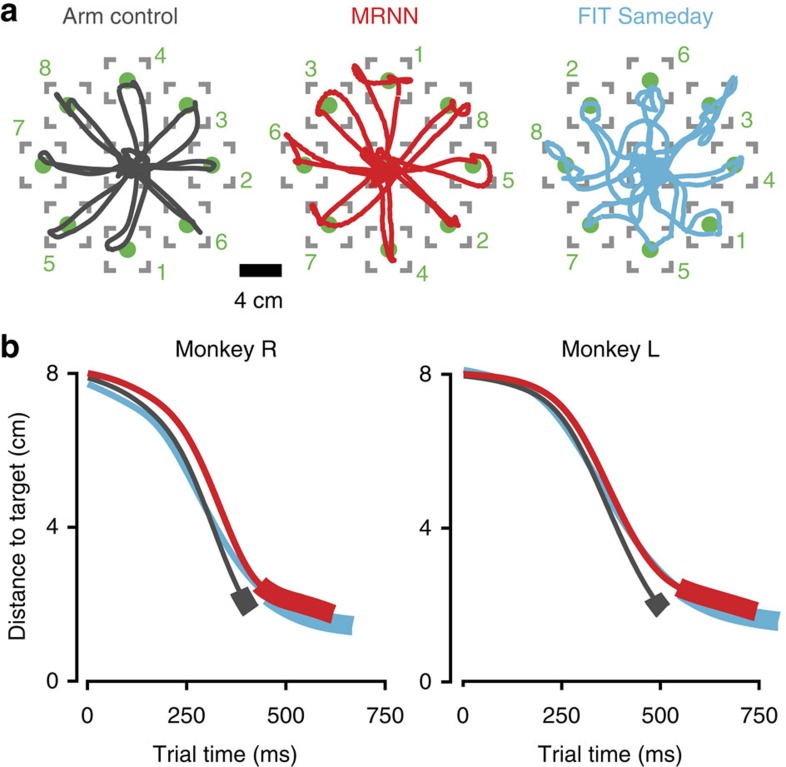
MRNN achieves high-performance under ‘ideal' conditions. (**a**) We compared cursor control using the MRNN (red) trained from many data sets up through the previous day to the FIT Sameday (blue) trained from data collected earlier the same day, without any artificial challenges (that is, no electrodes dropped or stale training data). Cursor trajectories are shown for eight representative and consecutive centre-out-and-back trials of the Radial 8 Task. Grey boxes show the target acquisition area boundaries, and the order of target presentation is denoted with green numbers. For comparison, cursor trajectories under arm control are shown in grey. From dataset R.2014.04.03. (**b**) Mean distance to target, across all Radial 8 Task trials under these favourable conditions, as a function of trial time using each cursor-control mode. Thickened portions of each trace correspond to ‘dial-in time', that is, the mean time between the first target acquisition and the final target acquisition. These MRNN and FIT Sameday data correspond to the drop 0 electrodes condition data in Fig. 3, and include 4,094 (3,278) MRNN trials and 4119 (3,305) FIT Sameday trials over 9 (8) experimental days in Monkey R (L).

Finally, we note that the MRNN's robustness to challenging recording conditions did not come at the cost of reduced performance under more ‘ideal' conditions, that is, without electrode dropping or stale training data. During the electrode-dropping experiments, we also evaluated the MRNN's closed-loop performance after being trained using several months' data up through the previous day. In this scenario, the MRNN enabled both monkeys to accurately and quickly control the cursor. Supplementary Movie 1 shows example cursor control using the MRNN. These data also allowed us to compare the MRNN's performance with that of a FIT Sameday decoder in back-to-back ‘AB AB' tests. Figure 5a shows representative cursor trajectories using each decoder, as well as under hand control. Figure 5b shows that across 9 experimental sessions and 4,000+ trials with each decoder, Monkey R acquired targets 7.3% faster with the MRNN (0.619±0.324 s mean±s.d. vs. 0.668±0.469 s, *P*=4.2e−6, rank-sum test). Monkey L acquired targets 10.8% faster with the MRNN (0.743±0.390 s versus 0.833±0.532 s, *P*=1.5e−3, rank-sum test) across 8 sessions and 2,500+ trials using each decoder. These online results corroborate the offline results presented in Fig. 2c; both show that an MRNN trained from many days' recording conditions outperforms the FIT Kalman filter trained from training data collected at the start of the experimental session.

A potential risk inherent to a computationally powerful decoder such as the MRNN is that it will overtrain to the task structure of the training data and fail to generalize to other tasks. Most of our MRNN training data were from arm reaches on a Radial 8 Task similar to the task used for evaluation (albeit with 50% further target distance). We therefore also tested whether the MRNN enabled good cursor control on the Random Target Task, in which the target could appear in any location in a 20 × 20 cm workspace (Supplementary Fig. 7). Monkey R performed the Random Target Task on two experimental sessions and averaged a 99.4% success rate, with mean distance-normalized time to target of 0.068 s cm^−1^. Monkey L performed one session of this task at a 100% success rate with mean normalized time to target of 0.075 s cm^−1^. To provide context for these metrics, we also measured Random Target Task performance using arm control. Monkey R's arm control success rate was 100%, with 0.055 s cm^−1^ mean normalized time to target, during the same experimental sessions as his MRNN Random Target Task data. Monkey L's arm control success rate was 97.7%, with 0.055 s cm^−1^ mean normalized time to target, during one session several days following his MRNN test.

## Discussion

We developed the MRNN decoder to help address a major problem hindering the clinical translation of BMIs: once trained, decoders can be quickly rendered ineffective due to recording condition changes. A number of complementary lines of research are aimed at making BMIs more robust, including improving sensors to record from more neurons more reliably (for example, ref. 44); decoding multiunit spikes^10^,^30^,^45^ or local field potentials^31^,^32^,^46^ that appear to be more stable control signals than single-unit activity; and using adaptive decoders that update their parameters to follow changing neural-to-kinematic mappings^4^,^10^,^20^,^21^,^22^,^23^,^24^,^25^,^26^,^27^,^28^,^29^,^47^. Here we present the MRNN as a proof-of-principle of a novel approach: build a fixed decoder whose architecture allows it to be inherently robust to recording condition changes based on the assumption that novel conditions have some similarity to previously encountered conditions.

We stress that all of these approaches are complementary in several respects. For example, a decoder that is inherently more robust to neural signal changes, such as the MRNN, would still benefit from improved sensors, could operate on a mix of input signal types including single- and multiunit spikes and field potentials, and is especially well positioned to benefit from decoder adaptation. When performance degrades due to recording condition changes, both supervised^10^,^21^,^22^,^23^,^25^,^27^,^29^ and unsupervised^4^,^20^,^24^,^26^ adaptive decoders need a period of time in which control is at least good enough that the algorithm can eventually infer the user's intentions and use these to update its neural-to-kinematic model. Improved robustness may ‘buy enough time' to allow the decoder's adaptive component to rescue performance without interrupting prosthesis use. Here we have demonstrated the MRNN's advantages over a state-of-the-art static decoder, but comparing this strategy both against and together with adaptive decoding remains a future direction.

We demonstrated the MRNN's robustness to two types of recording condition changes. These changes were chosen because they capture key aspects of the changes that commonly challenge BMI decoders during clinical use. The stale training data experiments showed that the MRNN was usable under conditions where the passage of time would typically require recalibration of conventional decoders such as the FIT-KF. We do not mean to suggest that in a clinical setting one would want to—or would often have to—use a BMI without any training data from the immediately preceding several months. Rather, we used this experimental design to model recording condition changes that can happen on the time scale of hours in human BMI clinical trials^13^. Possible reasons for the greater recording condition variability observed in human participants compared with non-human primates include: more movement of the array relative to the human brain due to larger cardiovascular pulsations and epidural space; greater variability in the state of the BMI user (health, medications, fatigue and cognitive state); and more electromagnetic interference from the environment. The MRNN can take advantage of having seen the effects of these sources of variability in previously accumulated data; it can therefore be expected to become more robust over time as it builds up a ‘library' of neural-to-kinematic mappings under different recording conditions.

The electrode-dropping experiments, which demonstrated the MRNN's robustness to an unexpected loss of high-importance electrodes, are important for two reasons. First, sudden loss of input signals (for example, due to a electrode connection failure^48^,^49^), is a common BMI failure mode that can be particularly disabling to conventional BMI decoders^50^. The MRNN demonstrates considerable progress in addressing this so-called ‘errant unit' problem. Second, these results demonstrate that the MRNN trained with artificially perturbed neural data can be relatively robust even to a recording condition change that has not been encountered in past recordings.

The MRNN's robustness did not come at the cost of diminished performance under more ideal conditions. This result is nontrivial given the robustness-focused decisions that went into its design (for example perturbing the input spike trains in the training set). Instead, we found that the MRNN was excellent under favourable conditions, slightly outperforming a state-of-the-art same day trained FIT-KF decoder. Taken together, these results demonstrate that the MRNN exhibits robustness to a variety of clinically relevant recording condition changes, without sacrificing peak performance. These advances may help to reduce the onerous need for clinical BMI users to collect frequent retraining data.

One disadvantage of this class of nonlinear decoders trained from large data sets, when compared with traditional linear decoders trained on smaller data sets, is the longer training time. In the present study, which we did not optimize for fast training, this took multiple hours. This could be substantially sped up by iteratively updating the decoder with new data instead of retraining *de novo* and by leveraging faster computation available with graphics processing units, parallel computing, or custom hardware. A second disadvantage of the MRNN is that it appears to require more training data to saturate its performance (Fig. 2b) compared with conventional methods, such as FIT-KF, that are trained from calibration data collected on the same day. We do not view this as a major limitation because the motivation for using the MRNN is to take advantage of accumulated previous recordings. Nonetheless, it will be valuable to compare the present approach with other decoder architectures and training strategies, which may yield similar performance and robustness while requiring less training data.

The MRNN decoder's robustness was due to the combination of a large training data corpus, deliberate perturbation of the training data and a computationally powerful architecture that was able to effectively learn this diverse training data. While it may seem obvious that successfully learning more training data is better, this is not necessarily true. Older data only help a decoder if some of these past recordings capture neural-to-kinematic relationships that are similar to that of the current recording condition. Our offline and closed-loop MRNN robustness results suggest that this was indeed the case for the two monkeys used in this study. While there are indications that this will also be true in human BMI studies^14^, validating this remains an important future question. The relevance of old data to present recording conditions also motivates a different robustness-enhancing approach: store a library of different past decoders and evaluate each to find a decoder well-suited for the current conditions (for example, ref. 10). However, since offline analyses are poor predictors of closed-loop performance^32^,^42^,^45^,^51^, this approach necessitates a potentially lengthy decoder selection process. Using a single decoder (such as the MRNN) that works across many recording conditions avoids switching-related downtime.

In addition to training with months of previous data, we improved the MRNN's robustness by intentionally perturbing the training neural data. In the present study, we applied random Gaussian firing rate scaling based on a general assumption that the decoder should be broadly robust to both global and private shifts in observed firing rates. This perturbation type proved effective, but we believe that this approach (called data augmentation in the machine learning community) can potentially be much more powerful when combined with specific modelling of recording condition changes that the experimenter wants to train robustness against. For example, data augmentation could incorporate synthetic examples of losing a particularly error-prone set of electrodes; recording changes predicted by models of array micro-movement or degradation; and perhaps even the predicted interaction between kinematics and changes in cognitive state or task context. We believe this is an important avenue for future research.

We view the success of our specific MRNN decoder implementation as a validation of the more general BMI decoder strategy of training a computationally powerful nonlinear decoder to a large quantity of data representing many different recording conditions. This past data need not have been collected explicitly for the purpose of training as was done in this study; neural data and corresponding kinematics from past closed-loop BMI use can also serve as training data^4^,^10^. It is likely that other nonlinear decoding algorithms will also benefit from this strategy, and that there are further opportunities to advance the reliability and performance of BMIs by starting to take advantage of these devices' ability to generate large quantities of data as part of their regular use.

## Methods

### Animal model and neural recordings

All procedures and experiments were approved by the Stanford University Institutional Animal Care and Use Committee. Experiments were conducted with adult male rhesus macaques (R and L, ages 8 and 18 years, respectively), implanted with 96-electrode Utah arrays (Blackrock Microsystems Inc., Salt Lake City, UT) using standard neurosurgical techniques. Monkeys R and L were implanted 30 months and 74 months before the primary experiments, respectively. Monkey R had two electrode arrays implanted, one in caudal dorsal premotor cortex (PMd) and the other in primary motor cortex (M1), as estimated visually from anatomical landmarks. Monkey L had one array implanted on the border of PMd and M1. Within the context of the simple point-to-point arm and BMI reach behaviour of this study, we observed qualitatively similar response properties between these motor cortical areas; this is consistent with previous reports of a gradient of increasing preparatory activity, rather than stark qualitative differences, as one moves more rostral from M1 (refs 52, 53, 54, 55, 56). Therefore, and in keeping with standard BMI decoding practices^6^,^8^,^10^,^24^,^38^,^40^,^46^, we did not distinguish between M1 and PMd electrodes.

Behavioural control and neural decode were run on separate PCs using the xPC Target platform (Mathworks, Natick, MA), enabling millisecond-timing precision for all computations. Neural data were initially processed by Cerebus recording system(s) (Blackrock Microsystems Inc., Salt Lake City, UT) and were available to the behavioural control system within 5±1 ms. Spike counts were collected by applying a single negative threshold, set to −4.5 times the root mean square of the spike band of each electrode. We decoded ‘threshold crossings', which contain spikes from one or more neurons in the electrode's vicinity, as per standard practice for intracortical BMIs^1^,^4^,^6^,^7^,^10^,^15^,^16^,^31^,^38^,^40^ because threshold crossings provide roughly comparable population-level velocity decode performance to sorted single-unit activity, without time-consuming sorting^30^,^45^,^57^,^58^,^59^, and may be more stable over time^30^,^45^. To orient the reader to the quality of the neural signals available during this study, Supplementary Note 1 provides statistics of several measures of electrodes' ‘tuning' and cross-talk.

### Behavioural tasks

We trained the monkeys to acquire targets with a virtual cursor controlled by either the position of the hand contralateral to the arrays or directly from neural activity. Reaches to virtual targets were made in a 2D frontoparallel plane presented within a 3D environment (MSMS, MDDF, USC, Los Angeles, CA) generated using a Wheatstone stereograph fused from two LCD monitors with refresh rates at 120 Hz, yielding frame updates within 7±4 ms (ref. 43). Hand position was measured with an infrared reflective bead tracking system at 60 Hz (Polaris, Northern Digital, Ontario, Canada). During BMI control, we allowed the monkey's reaching arm to be unrestrained^47^,^60^ so as to not impose a constraint upon the monkey that during BMI control he must generate neural activity that does not produce overt movement^61^.

In the Radial 8 Task the monkey was required to acquire targets alternating between a centre target and one of eight peripheral targets equidistantly spaced on the circumference of a circle. For our closed-loop BMI experiments, the peripheral targets were positioned 8 cm from the centre target. In hand-reaching data sets used for decoder training and offline decode, the targets were either 8 or 12 cm (the majority of data sets) from the centre. In much of Monkey L's training data, the three targets forming the upper quadrant were placed slightly further (13 and 14 cm) based on previous experience that this led to decoders with improved ability to acquire targets in that quadrant. To acquire a target, the monkey had to hold the cursor within a 4 cm × 4 cm acceptance window centred on the target for 500 ms. If the target was acquired successfully, the monkey received a liquid reward. If the target was not acquired within 5 s (BMI control) or 2 s (hand control) of target presentation, the trial was a failure and no reward was given.

Although the data included in this study span many months of each animal's research career, these data start after each animal was well-trained in performing point-to-point planar reaches; day-to-day variability when making the same reaching movements was modest. To quantify behavioural similarity across the study, we took advantage of having collected the same ‘Baseline Block' task data at the start of most experimental sessions: 171/185 monkey R days, 398/452 monkey L days. This consisted of ∼200 trials of arm-controlled Radial 8 Task reaches, with targets 8 cm from the centre. For each of these recording sessions, we calculated the mean hand *x* and *y* velocities (averaged over trials to/from a given radial target) throughout a 700 ms epoch following radial target onset for outward reaches and 600 ms following centre target onset for inward reaches (inward reaches were slightly faster). We concatenated these velocity time series across the 8 different targets, producing 10,400 ms *x* velocity and *y* velocity vectors from each recording session. Behavioural similarity between any two recording sessions was then measured by the Pearson correlation between the data sets' respective *x* and *y* velocity vectors. Then, the two dimensions' correlations were averaged to produce a single-correlation value between each pair of sessions. These hand velocity correlations were 0.90±0.04 (mean±s.d. across days) for monkey R, and 0.91±04 for monkey L.

We measured closed-loop BMI performance on the Radial 8 Task using two metrics. Target acquisition rate is the number of peripheral targets acquired divided by the duration of the task. This metric holistically reflects cursor-control ability because, unlike time to target, it is negatively affected by failed trials and directly relates to the animal's rate of liquid reward. Targets per minute is calculated over all trials of an experimental condition (that is, which decoder was used) and therefore yields a single measurement per day/experimental condition. Across-days distributions of a given decoder's targets per minute performance were consistent with a normal distribution (Kolmogorov-Smirnov test), justifying our use of paired *t-*tests statistics when comparing this metric. This is consistent with the measure reflecting the accumulated outcome of many hundreds of random processes (individual trials). As a second measure of performance that is more sensitive when success rates are high and similar between decoders (such as the ‘ideal' conditions where we presented no challenges to the decoders), we compared times to target. This measure consists of the time between when the target appeared and when the cursor entered the target acceptance window before successfully acquiring the target, but does not include the 500 ms hold time (which is constant across all trials). Times to target are only measured for successful trials to peripheral targets, and were only compared when success rates were not significantly different (otherwise, a poor decoder with a low success rate that occasionally acquired a target quickly by chance could nonsensically ‘outperform' a good decoder with 100% success rate but slower times to target). Because these distributions were not normal, we used the Mann–Whitney–Wilcoxon rank-sum tests when comparing two decoders' times with target.

In the Random Target Task each trial's target appeared at a random location within a 20 cm × 20 cm region centred within a larger workspace that was 40 × 30 cm. A new random target appeared after each trial regardless of whether this trial was a success or a failure due to exceeding the 5 s time limit. The target location randomization enforced a rule that the new target's acceptance area could not overlap with that of the previous target. Performance on the Random Target Task was measured by success rate (the number of successfully acquired targets divided by the total number of presented targets) and the normalized time to target. Normalized time to target is calculated for successful trials following another successful trial, and is the duration between target presentation and target acquisition (not including the 500 ms hold time), divided by the straight-line distance between this target's centre and the previously acquired target's centre^62^.

### Decoder comparison experiment design

All offline decoding comparisons between MRNN and FIT-KF were performed using test data that were held out from the data used to train the decoders. Thus, although the MRNN has many more parameters than FIT-KF, both of these fundamentally different algorithm types were trained according to best practices with matched training and test data. This allows their performance to be fairly compared. Decode accuracy was measured as the square of the Pearson's correlation coefficient between true and decoded hand endpoint velocity in the fronto-parallel plane.

When comparing online decoder performance using BMI-controlled Radial 8 Target or Random Target Tasks, the decoders were tested using an interleaved block-set design in which contiguous ∼200 trial blocks of each decoder were run followed by blocks of the next decoder, until the block-set comprising all tested decoders was complete and the next block-set began. For example, in the electrode-dropping experiments (Fig. 3), this meant an ‘AB AB' design where A could be a block of MRNN trials and B could be a block of FIT Sameday trials. For the stale training data experiments (Fig. 4), an ‘ABCD ABCD ABCD… ' design was used to test the four different decoders. When switching decoders, we gave the monkey ∼20 trials to transition to the new decoder before starting ‘counting' performance in the block; we found this to be more than sufficient for both animals to adjust. For electrode-dropping experiments, the order of decoders within each block-set was randomized across days. For stale training data experiments, where several decoders often performed very poorly, we manually adjusted the order of decoders within block-sets so as to keep the monkeys motivated by alternating what appeared to be more and less frustrating decoders. All completed blocks were included in the analysis. Throughout the study, the experimenters knew which decoder was in use, but all comparisons were quantitative and performed by the same automated computer program using all trials from completed blocks. The monkeys were not given an overt cue to the decoder being used.

During online experiments, we observed that when a decoder performed extremely poorly, such that the monkey could not reliably acquire targets within the 5 s time limit, the animal stopped performing the task before the end of the decoder evaluation block. To avoid frustrating the monkeys, we stopped a block if the success rate fell below 50% after at least 10 trials. This criterion was chosen based on pilot studies in which we found that below this success rate, the monkey would soon thereafter stop performing the task and would frequently refuse to re-engage for a prolonged period of time. Our interleaved block design meant that each decoder was tested multiple times on a given experimental session, which in principle provides the monkey multiple attempts to finish a block with each decoder. In practice, we found that monkeys could either complete every block or no blocks with a given decoder, and we refer to decoders that could not be used to complete a block as having failed. The performance of these decoders was recorded as 0 targets per minute for that experimental session. The exception to the above was that during an electrode-dropping experiment session, we declared both FIT-KF Sameday and MRNN as having failed for a certain number of electrodes dropped if the monkey could not complete a block with either decoder. That is, we did not continue with a second test of both (unusable) decoders as per the interleaved block design, because this would have unduly frustrated the animal.

We performed this study with two monkeys, which is the conventional standard for systems neuroscience and BMI experiments using a non-human primate model. No monkeys were excluded from the study. We determined how many experimental sessions to perform as follows. For all offline analyses, we examined the dates of previous experimental sessions with suitable arm reaching data and selected sets of sessions with spacing most appropriate for each analysis (for example, closely spaced sessions for Fig. 2b, all of the available data for Fig. 2c, two clusters with a gap for stale training analyses). All these predetermined sessions were then included in the analysis. For the stale training data experiments (Fig. 4), the choice of two gaps with three test days each was pre-established. For the electrode-dropping experiments (Fig. 3), we did not know *a priori* how electrode dropping would affect performance and when each decoder would fail. We therefore determined the maximum number of electrodes to drop during the experiment and adjusted the number of sessions testing each drop condition during the course of experiments to comprehensively explore the ‘dynamic range' across which decoder robustness appeared to differ. For both of these experiments, during an experimental session additional block-sets were run until the animal became satiated and disengaged from the task. We did not use formal effect size calculations to make data sample size decisions, but did perform a variety of experiments with large numbers of decoder comparison trials (many tens of thousands) so as to be able to detect substantial decoder performance differences. For secondary online experiments (Supplementary Figs 3 and 7), which served to support offline analyses (Supplementary Fig. 3) or demonstrate that the MRNN could acquire other target locations (Supplementary Fig. 7), we chose to perform only 1–3 sessions per animal in the interest of conserving experimental time.

### Neural decoding using an MRNN

At a high level, the MRNN decoder transforms inputs **u**(*t*), the observed spike counts on each electrode at a particular time, into a cursor position and velocity output. This is accomplished by first training the artificial recurrent neural network; that is, adjusting the weights of an artificial recurrent neural network such that when the network is provided a time series of neural data inputs, the data kinematic outputs can be accurately ‘read out' from this neural network's state. The rest of this section will describe the architecture, training and use of the MRNN for the purpose of driving a BMI.

The generic recurrent network model is defined by an *N*-dimensional vector of activation variables, **x**, and a vector of corresponding ‘firing rates', **r**=tanh **x**. Both **x** and **r** are continuous in time and take continuous values. In the standard RNN model, the input affects the dynamics as an additive time-dependent bias in each dimension. In the MRNN model, the input instead directly parameterizes the artificial neural network's recurrent weight matrix, allowing for a multiplicative interaction between the input and the hidden state. One view of this multiplicative interaction is that the hidden state of the recurrent network is selecting an appropriate decoder for the statistics of the current data set. The equation governing the dynamics of the activation vector is of the form suggested in ref. 35, but adapted in this study to continuous time to control the smoothness to MRNN outputs,


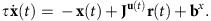


The *N* × *N* × |**u**| tensor **J**^**u**(*t*)^ describes the weights of the recurrent connections of the network, which are dependent on the *E*-dimensional input, **u**(*t*). The symbol |**u**| denotes the number of unique values **u**(*t*) can take. Such a tensor is unusable for continuous valued **u**(*t*) or even discrete valued **u**(*t*) with prohibitively many values. To make these computations tractable, the input is linearly combined into *F* factors and **J**^**u**(*t*)^ is factorized^35^ according to the following formula:


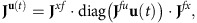


where **J**^*xf*^ has dimension *N* × *F*, **J**^*fu*^ has dimension *F* × *E*, **J**^*fx*^ has dimension *F* × *N*, and diag(**v**) takes a vector, **v**, and returns a diagonal matrix with **v** along the diagonal. One can directly control the complexity of interactions by choosing *F*. In addition, the network units receive a bias **b**^*x*^. The constant *τ* sets the time scale of the network, so we set *τ* in the physiologically relevant range of hundreds of milliseconds. The output of the network is read out from a weighted sum of the network firing rates plus a bias, defined by the equation


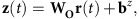


where **W**_**o**_ is an *M* × *N* matrix, and **b**^*z*^ is an *M*-dimensional bias.

### MRNN training

We began decoder training by instantiating MRNNs of network size *N*=100 (monkey R) and *N*=50 (monkey L) with *F*=*N* in both cases (see Table 1 for all MRNN parameters). For monkey R, who was implanted with two multielectrode arrays, *E*=192, while for monkey L with one array, *E*=96. The non-zero elements of the non-sparse matrices **J**^*xf*^,**J**^*fu*^,**J**^*fx*^ are drawn independently from a Gaussian distribution with zero mean and variance *g*_*xf*_/*F*,*g*_*fu*_/*E*, and *g*_*fx*_/*N*, with *g*_*xf*_,*g*_*fu*_, and *g*_*fx*_ set to 1.0 in this study. The elements of **W**_**o**_ are initialized to zero, and the bias vectors **b**^*x*^ and **b**^*z*^ are also initialized to 0.

The input **u**(*t*) to the MRNN (through the matrix **J**^**u**(*t*)^) is the vector of binned spikes at each time step. Concatenating across time in a trial yields training data matrix, **U**^*j*^, of binned spikes of size *E* × *T*^*j*^, where *T*^*j*^ is the number of times steps for the *j*th trial. Data from five consecutive actual monkey-reaching trials are then concatenated together to make one ‘MRNN training' trial. The first two actual trials in an MRNN training trial were used for seeding the hidden state of the MRNN (that is, not used for learning), whereas the next three actual trials were used for learning. With the exception of the first two actual trials from a given recording day, the entire set of actual trials are used for MRNN learning by incrementing the actual trial index that begins each training trial by one.

The parameters of the network were trained offline to reduce the averaged squared error between the measured kinematic training data and the output of the network, **z**(*t*). Specifically, we used the Hessian-Free (HF) optimization method^36^,^63^ for RNNs (but adapted to the continuous-time MRNN architecture). HF is an exact second order method that uses back-propagation through time to compute the gradient of the error with respect to the network parameters. The set of trained parameters is {**J**^*xf*^,**J**^*fu*^,**J**^*fx*^,**b**^*x*^,**W**_**o**_,**b**^*z*^}. The HF algorithm has three critical parameters: the minibatch size; the initial lambda setting; and the max number of conjugate-gradient iterations. We set these parameters to one-fifth the total number of trials, 0.1 and 50, respectively. The optimizations were run for 200 steps and a snapshot of the network was saved every 10 steps. Among these snapshots, the network with the lowest cross-validation error on held-out data was used in the experiment.

We independently trained two separate MRNN networks to each output a 2D (*M*=2) signal, **z**(*t*). The first network learned to output the normalized hand position through time in both the horizontal (*x*) and vertical (*y*) spatial dimensions. The second MRNN learned to output the hand velocity through time, also in the *x* and *y* dimensions. As training data for the velocity decoder, we calculated hand velocities from the hand positions numerically using central differences.

In this study, we trained a new MRNN whenever adding new training data; this allowed us to verify that the training optimization consistently converged to a high-quality decoder. However, it is easy to iteratively update an MRNN decoder with new data without training from scratch. By adding the new data to the training corpus and using the existing decoder weights as the training optimization's initial conditions, the MRNN will more rapidly converge to a new high-quality decoder.

### Training an MRNN with many data sets and perturbed inputs

A critical element of achieving both high performance and robustness in the MRNN decoder was training the decoder using data from many previous recording days spanning many months. When training data sets included data from >1 day, we randomly selected a small number of trials from each day for a given minibatch. In this way, every minibatch of training data sampled the input distributions from all training days.

A second key element of training robustness to recording condition changes was a form of data augmentation in which we intentionally introduced perturbations to the neural spike trains that were used to train the MRNN. The concatenated input, 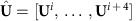 was perturbed by adding and removing spikes from each electrode. We focus on electrode *c* of the *j*th training trial, that is, a row vector of data 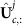. Let the number of actual observed spikes in 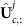 be 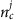. This number was perturbed according to


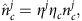


where both *η*^*j*^ and *η*_*c*_ are Gaussian variables with a mean of one and s.d. of *σ*_trial_ and *σ*_electrode_, respectively. Conceptually, *η*^*j*^ models a global firing rate modulation across all electrodes of the array (for example, array movement and arousal), while *η*_*c*_ models electrode by electrode perturbations such as electrode dropping or moving baselines in individual neurons. If 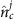 was <0 or >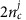, it was resampled, which kept the average number of perturbed spikes in a given electrode and training trial roughly equal to the average number of true (unperturbed) spikes in the same electrode and training trial. Otherwise, if 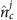 was greater than 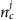, then 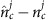 spikes were added to random time bins of the training trial. If 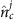 was less than 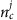, then 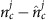 spikes were randomly removed from time bins of the training trial that already had spikes. Finally, if 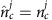, nothing was changed.

The process of perturbing the binned spiking data occurred anew on every iteration of the optimization algorithm, that is, in the HF algorithm, the perturbation 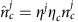 occurs after each update of the network parameters. Note that these input data perturbations were only applied during MRNN training; when the MRNN was used for closed-loop BMI control, true neural spike counts were provided as inputs. Supplementary Figure 3 shows the closed-loop control quality difference between the MRNN trained with and without this data augmentation. Our data augmentation procedure is reminiscent of dropout^64^, however our data perturbations are tailored to manage the nonstationarities in data associated with BMI.

### Controlling a BMI cursor with MRNN output

Once trained, the MRNNs were compiled into the embedded real-time operating system and run in closed-loop to provide online BMI cursor control. The decoded velocity and position were initialized to 0, as was the MRNN hidden state. Thereafter, at each decode time step the parallel pair of MRNNs received binned spike counts as input and had their position and velocity outputs blended to yield a position estimate. This was used to update the drawn cursor position. The on-screen position that the cursor moves to during BMI control, *d*_*x*_(*t*),*d*_*y*_(*t*), is defined by


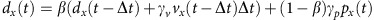



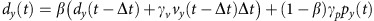


where *v*_*x*_, *v*_*y*_, *p*_*x*_, *p*_*y*_ are the normalized velocity and positions in the *x* and *y* dimensions and *γ*_*v*_,*γ*_*p*_ are factors that convert from the normalized velocity and position, respectively, to the coordinates of the virtual-reality workspace. The parameter β sets the amount of position versus velocity decoding and was set to 0.99. In effect, the decode was almost entirely dominated by velocity, with a slight position contribution to stabilize the cursor in the workplace (that is, offset accumulated drift). Note that when calculating offline decode accuracy (Fig. 2), we set β to 1 to more fairly compare the MRNN to the FIT-KF decoder, which decodes velocity only.

We note that although (1) the MRNN's recurrent connections mean that previous inputs affect how subsequent near-term inputs are processed, and (2) our standard procedure was to retrain the MRNN with additional data after each experimental session, the MRNN is *not* an ‘adaptive' decoder in the traditional meaning of the term. Its parameters are fixed during closed-loop use, and therefore when encountering recording condition changes, the MRNN cannot ‘learn' from this new data to update its neural-to-kinematic mappings in the way that adaptive decoders do (for example, refs 4, 24, 27). Insofar as its architecture and training regime make the MRNN robust to input changes, this robustness is ‘inherent' rather than ‘adaptive.'

### Neural decoding using a FIT-KF

We compared the performance of the MRNN with FIT-KF^40^. The FIT-KF is a Kalman filter where the underlying kinematic state, **z**(*t*), comprises the position and velocity of the cursor as well as a bias term. Observations of the neural binned spike counts, **y**(*t*), are used to update the kinematic state estimate. With Δ*t* denoting bin width (25 ms in this study), the FIT-KF assumes the kinematic state gives rise to the neural observations according to the following linear dynamical system:


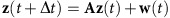



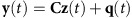


where **w**(*t*) and **q**(*t*) are zero-mean Gaussian noise with covariance matrices **W** and **Q**, respectively. The Kalman filter is a recursive algorithm that estimates the state **z**(*t*) using the current observation **y**(*t*) and the previous state estimate **z**(*t*−Δ*t*). Previous studies have used such decoders to drive neural cursors (for example refs 5, 38, 65).

The parameters of this linear dynamical system, **A,W,C,Q,** are learned in a supervised manner from hand reach training data using maximum-likelihood estimation, further described in refs 6, 66. The FIT-KF then incorporates two additional innovations. First, it performs a rotation of the training kinematics using the assumption that at every moment in time, the monkey intends to move the cursor directly towards the target. Second, it assumes that at every time step, the monkey has perfect knowledge of the decoded position via visual feedback. This affects Kalman filter inference in two ways: first, the covariance of the position estimate in Kalman filtering is set to 0; and second, the neural activity that is explainable by the cursor position is subtracted from the observed binned spike counts. These innovations are further described in refs 6, 40.

### Mutual information for determining electrode-dropping order

When testing the decoders' robustness to unexpected electrode loss, we determined which electrodes to drop by calculating the mutual information between each electrode's binned spike counts and the reach direction. This metric produced a ranking of electrodes in terms of how statistically informative they were of the reach direction; importantly, this metric is independent of the decoder being used. Let *p* denote the distribution of an electrode's binned firing rates, *y* denote the binned spike counts lying in a finite set *Y* of possible binned spike counts, *M* denote the number of reach directions and *x*_*j*_ denote reach direction *j*. The set *Y* comprised {0,1,2,3,4,5+} spike counts, where any spike counts greater than or equal to 5 were counted towards the same bin (‘5+', corresponding to an instantaneous firing rate of 250 Hz in a 20 ms bin). We calculated the entropy of each electrode,


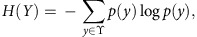


as well as its entropy conditioned on the reach direction


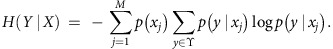


From these quantities, we calculated the mutual information between the neural activity and the reach direction as *I*_drop_(*X*;*Y*)=*H*(*Y*)−*H*(*Y*|*X*). We dropped electrodes in order from highest to lowest mutual information.

### Principal angles of neural subspaces analysis

For a parsimonious scalar metric of how similar patterns of neural activity during reaching were between a given pair of recording days (used in Supplementary Fig. 1), we calculated the minimum principal angle between the neural subspaces of each recording day. We defined the neural subspace on a recording day as the top *K* principal components of the neural coactivations. Put more simply, we asked how similar day *i* and day *j*'s motifs of covariance between electrodes' activity were during arm reaching. Specifically, we started with a matrix **Y**_*i*_ from each day *i* consisting of neural activity collected while the monkey performed ∼200 trials of a Radial 8 Task (8 cm distance to targets) using arm control; this task has been run at the start of almost every experimental session conducted using both monkeys R and L since array implantation. **Y**_*i*_ is of dimensionality *E* × *T*, where *E* is the number of electrodes and *T* is the number of non-overlapping 20 ms bins comprising the duration of this task. We next subtracted from each row of **Y**_*i*_ that electrode's across-days mean firing rate (we also repeated this analysis without across-days mean subtraction and observed qualitatively similar results, not shown). To obtain the principal components, we performed eigenvalue decomposition on the covariance matrix 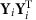 (note, **Y**_*i*_ is zero mean), and defined the matrix **V**_*i*_ as the first *K* eigenvectors. **V**_*i*_ had dimensions *E* × *K*, where each column *k* is the vector of principal component coefficients (eigenvector) corresponding to the *k*th largest eigenvalue of the decomposition. Supplementary Figure 1 was generated using *K*=10, that is, keeping the first 10 PCs, but the qualitative appearance of the data were similar when *K* was varied from 2 to 30 (not shown). Finally, the difference metric between days *i* and *j* was computed as the minimum of the *K* subspace angles between matrices **V**_*i*_ and **V**_*j*_. Subspace angles were computed using the *subspacea* MATLAB function^67^.

### Data availability

All relevant data and analysis code can be made available by the authors on request.

## Additional information

**How to cite this article:** Sussillo, D. *et al*. Making brain–machine interfaces robust to future neural variability. *Nat. Commun.* **7,** 13749 doi: 10.1038/ncomms13749 (2016).

**Publisher's note:** Springer Nature remains neutral with regard to jurisdictional claims in published maps and institutional affiliations.

## Supplementary Material

Supplementary Information Supplementary Figures 1-7 and Supplementary Note 1.

Supplementary Movie 1 The MRNN was trained using reaching data from 125 recording sessions up through the previous day. The video shows a continuous 90 seconds of monkey R using this decoder to perform the Radial 8 Task. He controls the white cursor and acquires the green target (which turns blue during the 500 ms target hold period). Dataset R.2014.04.03. This is a portion of the data used to generate the drop 0 electrodes condition of Fig. 3.

Supplementary Movie 2 During the experiment, the two decoders were evaluated in alternating blocks after the same 3 most important electrodes were dropped. Here we show a continuous 60 seconds of each decoder's closed-loop performance from consecutive blocks. The MRNN (right side) was trained using reaching data from 125 recording sessions up through the previous day, while the FIT Kalman filter (left side) was trained using reaching data from earlier that same day. Dataset monkey R.2014.04.03. This is a portion of the data used to generate the drop 3 electrodes condition of Fig. 3.

## Figures and Tables

**Figure 1:**
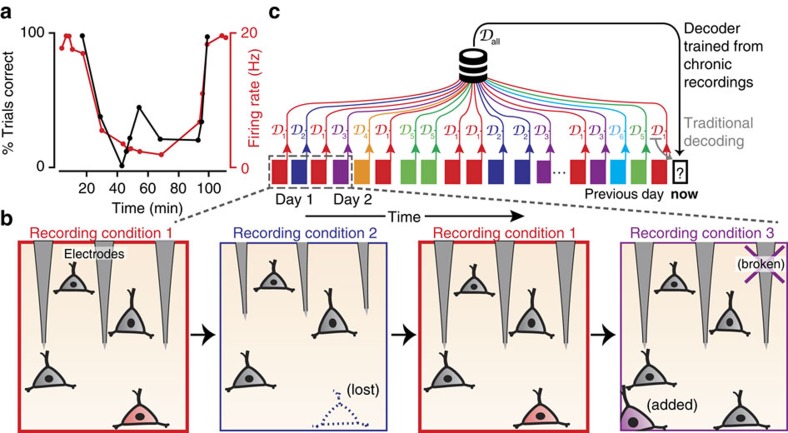
Strategy for training a decoder robust to recording condition changes. (**a**) Example data from a BMI clinical trial showing sudden decoder failure caused by a recording condition change. The black trace shows the participant's closed-loop performance over the course of an experiment using a fixed Kalman filter. An abrupt drop in performance coincides with a reduction in the observed firing rate (red trace) of a neuron with a high decoder weight. Both the neuron's firing rate and decoder performance spontaneously recover ∼40 min later. Adapted from Figure 7 of ref. 13. (**b**) A cartoon depicting one hypothetical cause of the aforementioned change: micro-motion of the electrodes leads to Recording Condition 2, in which spikes from the red-shaded neuron are lost. BMI recovery corresponds to a shift back to Condition 1. Over time, further changes will result in additional recording conditions; for example, Condition 3 is shown caused by a disconnected electrode and an additional neuron entering recording range. (**c**) Recording conditions (schematized by the coloured rectangles) will vary over the course of chronic intracortical BMI use. We hypothesize that oftentimes new conditions are similar to ones previously encountered (repeated colours). Typically, decoders are fit from short blocks of training data and are only effective under that recording condition (decoders 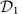, 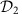, …). Consider the goal of training a decoder for use at time ‘now' (black rectangle on right). Standard practice is to use decoder 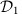 trained from the most recently available data (for example, from the previous day or the start of the current experiment). 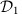 will perform poorly if the recording condition encountered differs from its training data. To increase the likelihood of having a decoder that will perform well given the current recording condition, we tested a new class of decoder, 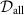, trained using a large collection of previous recording conditions.

**Table 1:** Network and training parameters used for the closed-loop MRNN BMI decoder.

	**Monkey R**	**Monkey L**
Δ*t*	20 ms	20–30 ms
*τ*	100 ms	100–150 ms
*N*	100	50
*F*	100	50
*σ*_trial_	0.045	0.045
*σ*_electrode_	0.3	0.3
*g*_*xf*_	1.0	1.0
*g*_*fu*_	1.0	1.0
*g*_*fx*_	1.0	1.0
*E*	192	96
Days of training data	82–129	189–230
Years spanned	1.59	2.77
Number of params in each MRNN	39502	9952
*β*	0.99	0.99

BMI, brain–machine interface; MRNN, multiplicative recurrent neural network.
