# Corrigendum: Making brain-machine interfaces robust to future neural variability

**DOI:** 10.1038/ncomms14490

**Published:** 2017-01-20

**Authors:** David Sussillo, Sergey D. Stavisky, Jonathan C. Kao, Stephen I. Ryu, Krishna V. Shenoy

Nature Communications 7: Article number: 13749; DOI: 10.1038/ncomms13749 (2016); Published: 12 13 2016; Updated: 01 20 2017

The financial support for this Article was not fully acknowledged. The Acknowledgements should have included the following:

This work was supported by the National Science Foundation Graduate Research Fellowship (J.C.K., S.D.S.); NSF IGERT 0734683 (S.D.S.); Christopher and Dana Reeve Paralysis Foundation (S.I.R. and K.V.S.); and the following to K.V.S.: Burroughs Welcome Fund Career Awards in the Biomedical Sciences, Defense Advanced Research Projects Agency Reorganization and Plasticity to Accelerate Injury Recovery N66001-10-C-2010, US National Institutes of Health Institute of Neurological Disorders and Stroke Transformative Research Award R01NS076460, US National Institutes of Health Director's Pioneer Award 8DP1HD075623-04, US National Institutes of Health Director's Transformative Research Award (TR01) from the NIMH #5R01MH09964703, and Defense Advanced Research Projects Agency NeuroFAST award from BTO #W911NF-14-2-0013.

