# Supplementary material for: Making brain–machine interfaces robust to future neural variability
Source: Nat Commun. 2016 Dec 13;7:13749. doi: 10.1038/ncomms13749 (PMC5159828; doi:10.1038/ncomms13749)
Supplement: Supplementary Information — Supplementary Figures 1-7 and Supplementary Note 1. [file ncomms13749-s1.pdf]

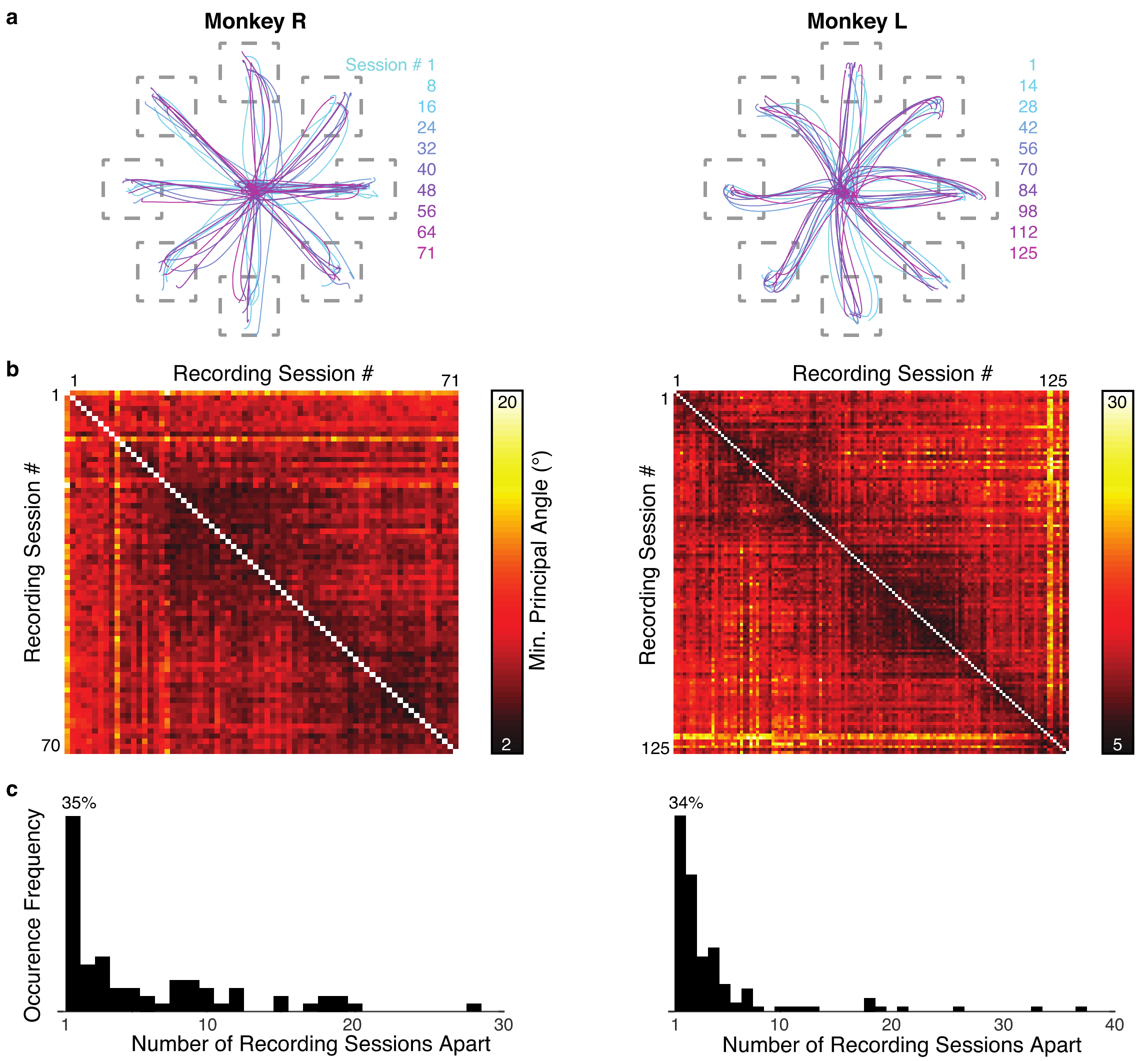

Supplementary Figure 1. For a given day, similar neural conditions can be found on some other day(s) Chronologically close days tend to have more similar neural recordings, but for a given day there are occasional similar recordings from more distant days. (**a**) To minimize the potential effect of behavioral variability on neural variability, we restricted this analysis to recording sessions with very consistent Radial 8 Target task behavior. Hand velocity correlations between all pairs of sessions within the included set were at least 0.9. Representative hand position traces (mean over trials towards each target) are shown for ten sessions spanning the months analyzed. (**b**) Between-day variability of the structure of neural activity recorded during reaches over the course of many months (71 recording sessions over a 658 day period in monkey R, and 125 sessions spanning 1003 days in monkey L; these correspond to a subset of the days included in Fig. 2c). The color at the intersection of row *i* and column *j* corresponds to how differently the observed neural activity covaried during recording sessions *i* and *j*. Specifically, we have plotted the minimum principal angle between subspaces spanned by the top 10 eigenvectors of each day's mean-activity-subtracted covariance matrix (see Methods). These 10 eigenvectors captured on average 51 (46)% of single-trial variance for monkeys R (L). Sharp "block" structure transitions typically correspond to a long (many weeks') interval between consecutive recording sessions. (**c**) Histograms showing the distribution, across each

monkey’s recordings, of how many recording sessions apart (either forward or back in time) we observed the most similar neural correlates of reaching as measured by minimum principal angle.

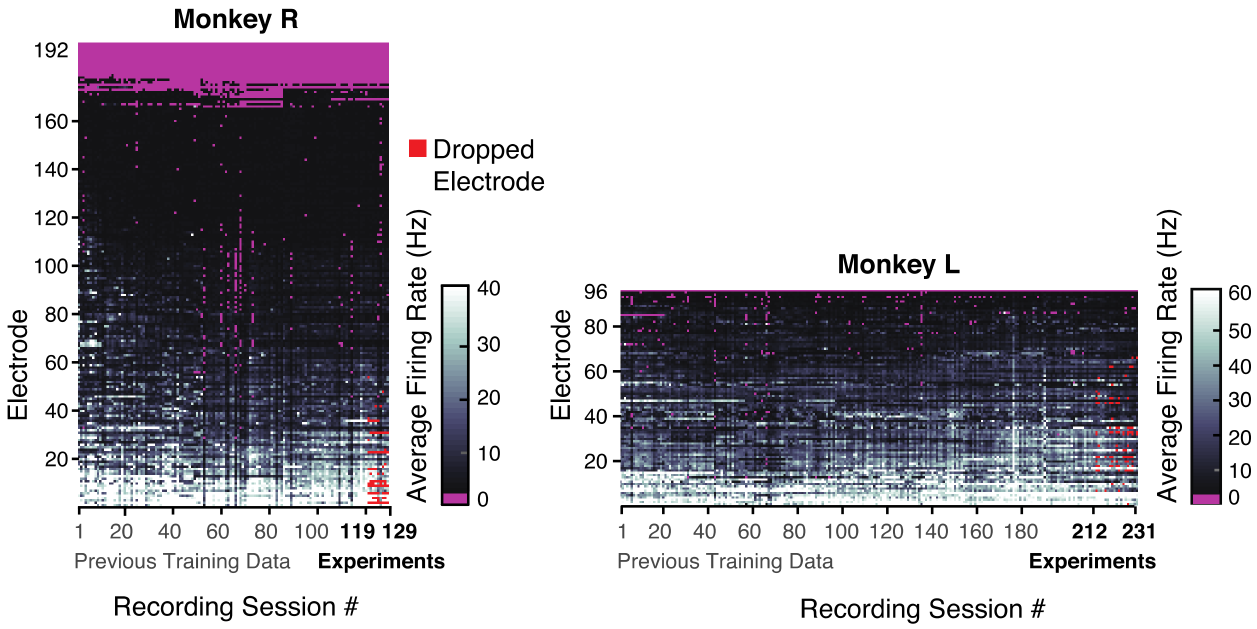

Supplementary Figure 2. Artificially dropped electrodes were active in the training data
These plots show each electrode's average firing rate during each dataset used to train the MRNN; electrodes are ordered by descending average firing rate across all recording sessions. Recording sessions numbered in gray were only used for training data. The electrode dropping experiments (Fig. 3) were conducted during the sessions numbered in black. Zero firing rates (i.e. non-functional electrodes) are shown in purple for emphasis, while electrodes selected for dropping on a particular day are shown in red (note that although on a given test session we evaluated different numbers of electrodes dropped, this plot shows each day's broadest dropped set). These dropped electrodes rarely recorded zero firing rates in the training data sessions, and the specific sets of dropped electrodes used to challenge the decoders never all had zero firing rates in the training data.

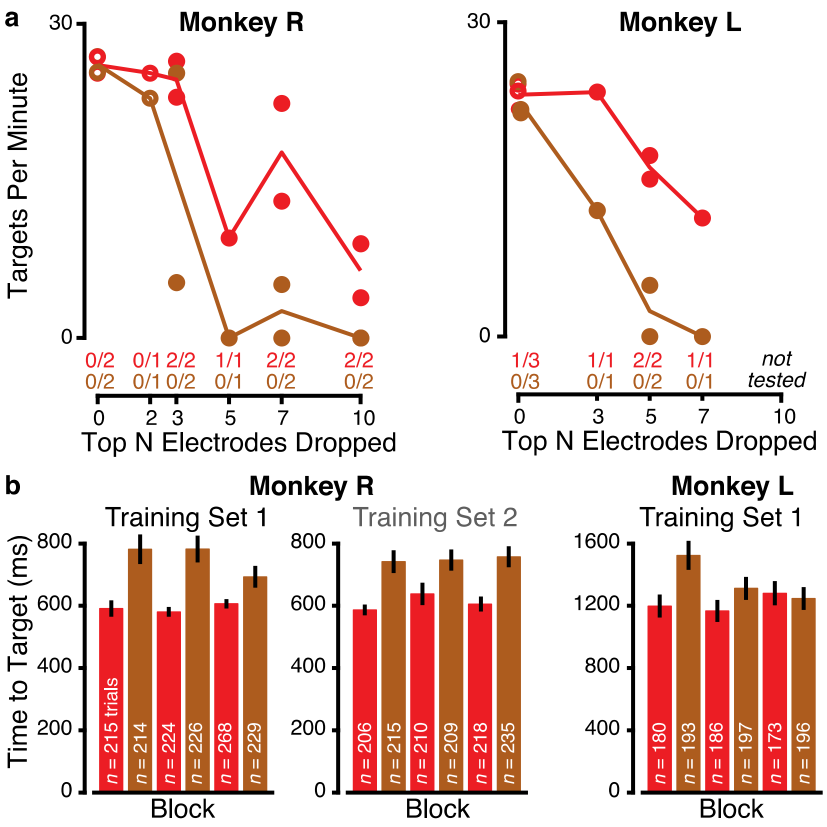

Supplementary Figure 3. Training data spike rate perturbations improve closed-loop MRNN robustness

(**a**) Robustness to electrode dropping. We evaluated the closed-loop BMI performance of the MRNN decoder trained with (red) and without (brown) the spike rate perturbations training data augmentation. Both decoders were evaluated on the same day with firing rates on varying numbers of the most informative electrodes set to zero (similar to Fig. 3). Each circle corresponds to a decoder's targets per minute performance on a given evaluation day. In total there were 3 sessions per monkey. Filled circles denote conditions where there was a significant within-session performance difference between the two decoders tested according to: $p < 0.05$ binomial test on success rate, followed, if success rate was not significantly different, by a more sensitive comparison of times to target ($p < 0.05$, rank-sum test). Fractions above the horizontal axis specify for how many of the individual evaluation days each decoder performed significantly better than the other. Trend lines show the across-sessions mean targets per minute performance for each decoder. The MRNN trained with perturbed firing rates outperformed the MRNN trained without data augmentation when encountering electrode-dropped neural input.

(**b**) Robustness to naturally occurring neural recording condition changes. MRNNs were trained without access to recent training data, as in the Fig. 4 stale training data experiments, either with (red) or without (brown) training data spike rate perturbations. We trained decoders from both of monkey R's stale training data periods and from monkey L's longer stale training data period. Closed-loop BMI performance using these decoders was then compared on the same evaluation day in alternating blocks. Bars show mean ± s.e.m. time to target for each block of trials (success rates using both training paradigms were close to 100%). The MRNN with spike rate perturbations had significantly faster times to target in monkey R ($p < 0.05$, rank-

sum test aggregating trials across blocks) and showed a trend of faster times to target in monkey L ($p = 0.066$). Datasets R.2014.03.21 & L.2014.04.04.

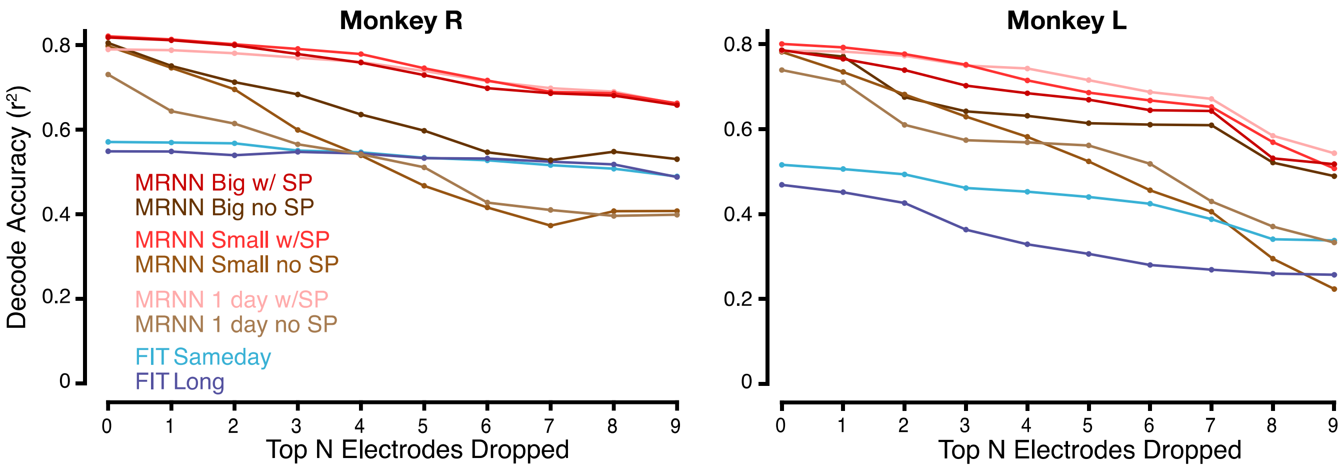

Supplementary Figure 4. Offline decoding to test robustness of training paradigms to electrode loss
We performed offline decoding analyses to test how each of the three main components of our method – the use of an MRNN architecture instead of a linear Kalman filter, the use of large training datasets, and the spike count perturbation data augmentation – contributed to improved robustness to unexpected loss of the most important electrodes, similar to the online tests shown in Figure 3. The results suggest that both the use of the MRNN architecture and training data augmentation contributed to the complete system's improved robustness to a novel recording condition consisting of electrode dropping.

We trained MRNNs with different quantities of data: 1 day of (held out) data from the test day, a 'Small' dataset consisting 10-13 days up to and including the test day, or a "Big" dataset of 40 – 100 days up to and including the test day, with ("w/SP") or without ("no SP") additional spike count perturbations during training. We also trained a FIT-KF Sameday decoder and a FIT-KF Long which used the same datasets as the MRNN Big datasets. We compared the offline decoding accuracy of each decoder as a function of the number of electrodes dropped, using the same electrode dropping order determination method as in the Figure 3 online experiments. We note that the relationship between offline decode r^2 and online performance is complicated, and therefore it is difficult to precisely predict online performance from offline r^2. Nevertheless, substantial differences in r^2 arising from different decoder interventions can be informative of each intervention's usefulness in online decoding. Three decoders were trained for each training paradigm using data from different periods of each monkey's research career; these decoders' training dates correspond to exactly the same as those in Supplementary Figure 6, and each decoder was tested on held out data from its last day of training. We averaged offline hand velocity reconstruction accuracy across each monkey's three testing days. We found that applying the spike count perturbation always increased MRNN robustness to electrode dropping (compiling r^2 across all SP vs all no SP decoders across all three test days, SP decoders performed better than no SP decoders, $p < 0.001$, signed-rank test). Note that when using spike perturbations, training with larger dataset sizes did not strongly affect performance or robustness to electrode dropping, since all MRNN's have 'seen' data collected on the same day as the withheld testing data.

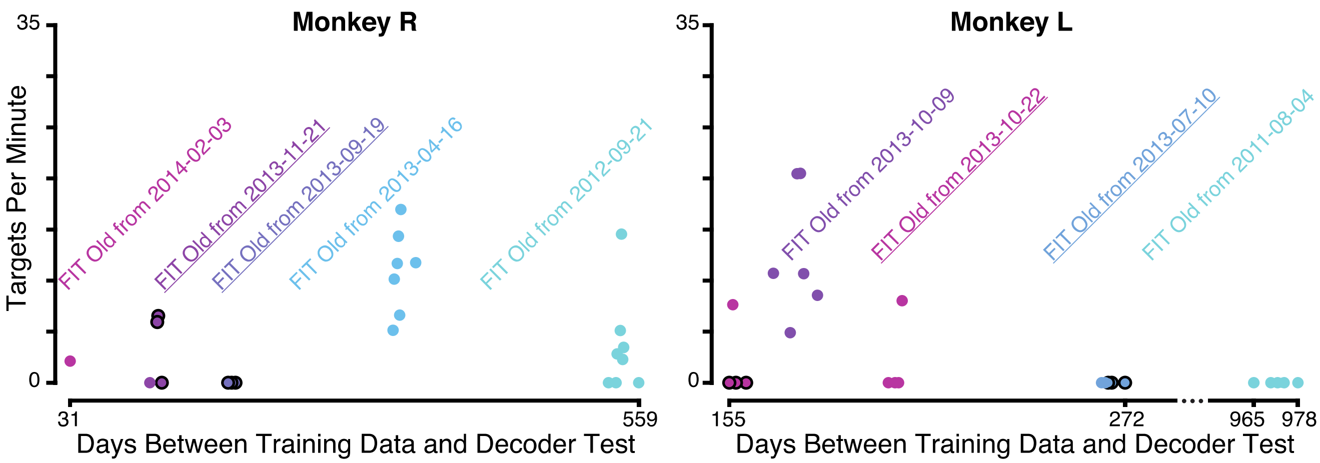

Supplementary Figure 5. Additional tests showing that FIT Old typically performs poorly

We investigated whether the reason that three of the four different FIT Old decoders tested in the main stale training data experiments (Fig. 4) failed was due to a particularly unlucky choice of FIT Olds. To better sample the closed-loop performance of FIT-KF decoders trained using old training data, we trained FIT Old decoders from 3 (monkey R) and 2 (Monkey L) additional arbitrarily chosen arm reaching datasets from the monkey's prior experiments. We evaluated all 5 (4) FIT Old decoders on a number of additional days over the course of the current study (8 total test days for monkey R, 13 total test days for monkey L). Each point shows the performance of a particular FIT Old decoder on one test day. Different days' evaluations of the same FIT Old decoder are shown in the same color. Black circle edges denote data points and underlines under decoder names denote decoders that are shared with Fig. 4.

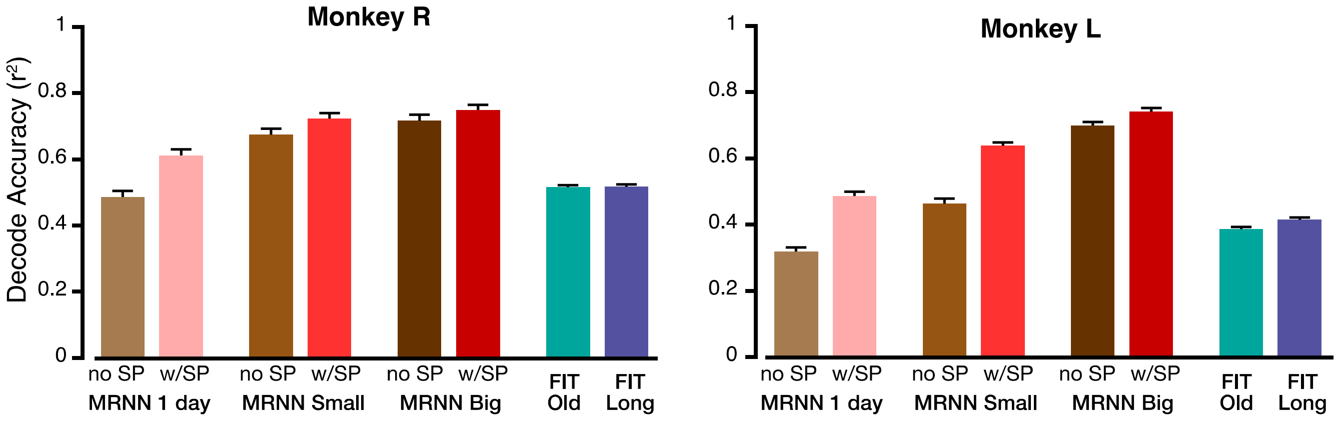

Supplementary Figure 6. Offline decoding to test robustness of training paradigms to neural variability
We performed offline decoding analyses to test how each of the three main components of our method – the use of an MRNN architecture instead of a linear Kalman filter, the use of large training datasets, and the spike count perturbation data augmentation – contributed to improved robustness to naturally occurring recording condition changes similar to the online 'stale' training data tests shown in Figure 4. We performed offline decoding evaluations across three different training data gaps and found that using more previously collected data and incorporating data augmentation both improved the MRNN's performance on the future test data. MRNNs trained with many datasets outperformed FIT-KFs trained using the same data ("FIT Long") or just the most recent data ("Fit Old"). These results suggest that all three components of the method contributed to the complete system's improved robustness.

(Left) Offline decode results for Monkey R. We performed an offline decode for 8 different types of decoders and aggregated each decoder's performance over the three gaps. The MRNN decoders were either trained with 1 day of data "1 day," a "Small" dataset (gap 1: 13 datasets, gaps 2 and 3: 10 datasets) or a "Big" dataset (gap 1: 40 datasets, gap 2: 44 datasets, gap 3: 37 datasets). The MRNN decoders were also either trained with no spike rate perturbations ("no SP") or with spike rate perturbations ("w/SP"). We also trained a FIT Old using the most recent dataset and a FIT Long which used the same datasets as the MRNN Big datasets. Gap 1 comprised training data from 2012-10-22 (YYYY-MM-DD) to 2013-04-19 and testing data from 2013-07-29 to 2013-11-21 (44 testing days). Gap 2 comprised training data from 2013-07-29 to 2013-11-21 and testing data from 2014-02-03 to 2014-04-07 (37 testing days). Gap 3 comprised training data from 2014-02-03 to 2014-04-07 and testing data from 2014-06-16 to 2014-08-19 (33 testing days). The bars show the mean ± s.d. performance of each training approach across all 3 gaps. We observed the same trends across individual gaps, with the MRNN Big w/SP decoder always achieving the best performance ($p < 0.01$, signed-rank test with every other decoder, all gaps).

(Right) Same for Monkey L. The Small datasets comprised 10 datasets (gap 1 and 2) or 11 datasets (gap 3), while the Big datasets comprised 103 datasets (gap 1), 105 datasets (gap 2), and 77 datasets (gap 3). Gap 1 comprised training data from 2010-03-04 to 2010-10-26 and testing data from 2011-01-17 to 2011-04-28 (51 testing days). Gap 2 comprised training data from 2011-01-18 to 2011-10-04 and testing data from 2012-04-02 to 2012-07-19 (51 testing days). Gap 3 comprised training data from 2012-04-02 to 2012-10-12 and testing data from 2013-01-26 to 2013-07-10 (37 testing days). Across individual gaps, the same trends showed were displayed, with the MRNN Big w/SP decoder always achieving the best performance ($p < 0.01$,

signed-rank test with every other decoder, all gaps, except in Gap 2 when comparing to MRNN Small w/SP, p = 0.1, and Gap 3 where it on average achieved a lower r^2 than MRNN Small w/SP and MRNN Big no SP).

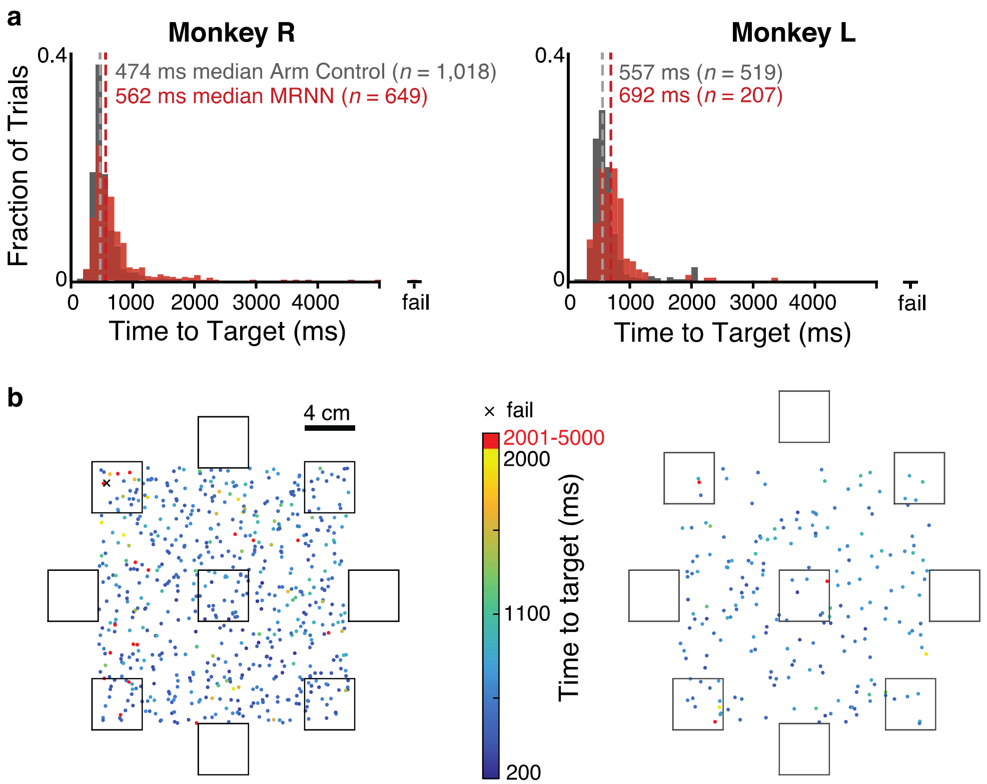

Supplementary Figure 7. Closed-loop MRNN decoder performance on the Random Target Task

Both monkeys were able to use the MRNN decoder to acquire targets across a broad workspace in which targets often appeared at locations that differed from the target locations dominating the training datasets.

(**a**) Histograms of Random Target Task times to target (time of final target entry minus target onset time, not including the 500 ms target hold period) using the MRNN decoder are shown in red. For comparison, histograms of performance on the same task using arm control are shown in gray.

(**b**) Task workspace plots showing the location of each Random Target Task trial's target during MRNN decoder evaluation. Each point corresponds to the center of one trial's target, and its color represents the time it took the monkey to acquire this target. The location of the one failed trial (for monkey R) is shown with a black 'x'. The acquisition area boundaries of the nine Radial 8 Task targets used for the majority of the training data are shown as black squares. Monkey R's data are aggregated across the two experimental sessions in which he performed this task. Monkey L's data are from one session.

Supplementary Note 1. Array recording quality measurements across this study

For task consistency, these analyses were restricted to those recording days when Baseline Block data was collected. Reach direction tuning was calculated as in [32]: for each recording day, we calculated each electrode's average firing rate over the course of each trial (analysis epoch: 200 to 600 ms after target onset) to yield a single data point per trial, and then grouped trials by target location. 69.0 ± 7.7 of monkey R's electrodes (mean ± s.d. across 171 recording days which had between 48 and 246 trials per day, with a mean of 111.0 trials) and 66.3 ± 12.3 of monkey L's electrodes (398 days, between 45 and 256 trials per day with a mean of 108.4 trials) exhibited significantly different firing rates when reaching to at least one of the eight different targets ($p < 0.01$, one-way ANOVA). These tuned electrodes' modulation range, defined here as the trial-averaged rates firing rate difference between reaches to the two targets evoking the highest and lowest rates, was 26.4 ± 5.9 Hz in monkey R monkey (mean ± s.d., averaged first across all electrode pairs in a given recording day, and then over days) and 23.8 ± 5.6 Hz in monkey L. We did not observe cross-talk between electrodes' threshold crossings, consistent with recording spiking activity from electrodes at least 400 μm apart: pairwise electrode cross-correlations, computed using the time-series of firing rates in consecutive non-overlapping 20 ms bins spanning a given day's Baseline Block, was 0.0089 ± 0.0021 in monkey R (mean ± s.d., averaged first across all electrode pairs in a given recording day, and then over days) and 0.0150 ± 0.0058 in monkey L.
